# The Effect of Evaluating Self's Emotions on Frontal Alpha Asymmetry

**DOI:** 10.1002/brb3.70419

**Published:** 2025-03-23

**Authors:** Masato Ito, Toru Takahashi, Yuto Kurihara, Rieko Osu

**Affiliations:** ^1^ Graduate School of Human Sciences Waseda University Tokorozawa Saitama Japan; ^2^ Faculty of Human Sciences Waseda University Tokorozawa Saitama Japan; ^3^ Japan Society for The Promotion of Science Chiyoda‐ku Tokyo Japan

**Keywords:** electroencephalography, emotion recognition, event‐related potential, frontal alpha asymmetry, measurement problem, self‐focused attention

## Abstract

**Purpose:**

In research to assess emotions from biometric signals, participants are asked to evaluate the emotions they subjectively experienced to confirm whether the assumed emotions were actually elicited. However, the evaluation of emotion may influence the biometric signals related to the emotion itself. While such evaluative processes may function as a form of emotion regulation, which is known to modulate emotional experiences, the neural mechanisms and effects of evaluation itself remain unclear. Specifically, the temporal dynamics of how these evaluations affect emotion‐related brain activity in electroencephalography (EEG) have not been investigated. Based on theories of emotional processing and self‐focused attention, we hypothesized that emotion evaluations would enhance emotional processing as reflected in frontal alpha asymmetry (FAA) through both immediate attentional effects and sustained self‐focused attention.

**Method:**

We measured a 29‐channel EEG in 40 healthy participants who were presented with unpleasant and highly arousing images. Participants were assigned to either an experimental group that performed the task with subjective evaluation followed by without subjective evaluation, or a control group that performed the task without subjective evaluation twice. This design allowed us to examine both immediate effects of evaluation and its lasting influence on subsequent emotional processing.

**Finding:**

The results revealed that FAA was significantly lower during emotional evaluation compared to conditions without subjective evaluation, particularly during stimulus processing (300–500 ms). This early modulation suggests that evaluation automatically engages attentional processes, may reflect enhanced negative emotional processing as well as the activation of behavioral inhibition system through self‐focused attention.

**Conclusion:**

This study demonstrates that the emotional evaluation procedure itself can significantly alter early emotion‐related brain activity, providing insights into how self‐focused emotional evaluation engages both emotional and motivational processes. These findings suggest the need for methodological reconsiderations in EEG emotion estimation studies.

## Graphical abstract

Frontal alpha asymmetry, a measure of relative frontal brain activity, was significantly lower in the condition in which participants rated their own emotions compared to the condition in which they did not, when presented with unpleasant and highly arousing images. This result indicates evaluating self‐emotion amplifies emotion‐related brain activity. This study provides a cautionary note regarding the use of such evaluations in EEG emotion estimation studies.

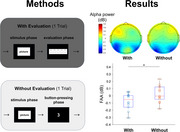

## 1 Introduction

Various studies have attempted to quantify human emotions using objective numerical values. Studies on human emotions have been performed using questionnaires in which respondent's' answers are based on categories of emotions (joy, sadness, anger, and happiness) or dimensions of emotions (pleasant–unpleasant, aroused–sedated) using a Likert scale. However, in recent years, more objective measurement methods using biological signals have been described (Shu et al. 2018). Electroencephalography (EEG) has been used to estimate emotion, and this approach has the advantage of allowing the prompt and objective observation of emotion, providing more information than peripheral signals (Alarcao and Fonseca 2019; Suhaimi et al. 2020). However, a considerable challenge of validity exists in the application of these techniques, as it has been reported that differences may exist between the experimental environment in which the data used to train the models are measured and the everyday environment to which the models are applied (Azari et al. 2020).

In the application of emotion recognition technology to everyday environments, the act of evaluating emotions is a necessary process that improves the validity and accuracy of recognition models. Evaluation, however, has been shown to alter the subjective emotional experience and emotion‐related brain activity in a top–down manner. For example, it has been confirmed that the act of reappraisal, a top–down controlled evaluation of a stimulus, can attenuate the emotional experience of the stimulus (Lazarus and Alfert 1964; Ochsner and Gross 2005). This reappraisal has been applied clinically to regulate emotional responses. It has also been observed that this reappraisal alters EEG indicators related to emotions (Hajcak and Nieuwenhuis 2006). These effects of evaluation can be understood within the broader framework of emotion regulation, where various cognitive processes serve to modulate emotional experiences and their neural bases through multiple temporal stages (Gross 1998). Indeed, even apparently simple evaluative processes may enhance emotional processing through increased self‐focused attention, as they involve attention to and reflection on one's emotional state (Füstös et al. 2013). Thus, evaluation is an important factor that determines emotional experience and may also alter emotional processing itself through both immediate attentional enhancement and sustained self‐focused attention.

Recent studies have extended these findings on the regulatory effects of evaluation by examining how the evaluation of stimuli itself influences brain activity. In studies that investigate the mechanism of “attention,” one of the methods used to induce attention to an emotional stimulus is to require the participants to evaluate the emotional aspect of the presented stimulus (Chun et al. 2011; Keller 2011). A comparison of trials with and without evaluation for olfactory pleasant/neutral/unpleasant stimuli revealed that evaluation for the emotion that the stimulus possesses modulates attention to the stimulus, which manifests as amplification of event‐related potential (ERP) P300 on EEG (Singh et al. 2019). In addition, functional magnetic resonance imaging (fMRI) studies of emotion have confirmed that emotion evaluation amplifies activity of emotion‐related brain areas, such as the amygdala (Lee and Siegle 2012). However, the effect of evaluating self‐emotion on EEG remains unclear.

In general, the temporal resolution of EEG is higher than that of fMRI and allows for the identification of very narrow temporal changes (Hennig et al. 2003). This temporal precision is particularly valuable in understanding emotion regulation processes, as different regulatory mechanisms operate on distinct time scales: attention‐related P300 emerges around 300–500 ms poststimulus, while emotion‐related LPP typically manifests from 500 ms onwards, with regulatory effects observed during 1000–2000 ms (Hajcak et al. 2010; Thiruchselvam et al. 2011). Self‐emotion evaluation is the process that occurs after stimuli are perceived. The temporal dynamics of how evaluation affects emotional processing remain unclear, particularly whether it influences early attentional processes (300–500 ms) or later emotional processing beyond 500 ms. The high temporal resolution of EEG, unlike fMRI, allows us to determine whether evaluation affects early attentional processes or later regulatory mechanisms. Therefore, in this study, we examined whether anticipation of emotional evaluation modulates emotion‐related EEG during stimulus processing, focusing on both early attentional processes and subsequent emotional processing.

This study also seeks to provide neuroscientific evidence of self‐focused attention, which directs attention to one's own emotions and internal changes. Muta and Koshikawa (2015) reported that pre‐mood measurement may influence the reaction time of a subsequent emotion word discrimination task. They describe that prior mood measurement elicited self‐focused attention that caused a delayed response to the stimulus. Although this self‐focused attention has been shown to boost emotion subjectively (Scheier and Carver 1977), from a neuroscientific perspective, the underlying brain activity is not clearly understood. According to the process model of emotion regulation (Gross 1998), attentional deployment as a form of emotion regulation typically functions to control emotions by diverting attention away from emotional aspects. However, attending to internal states without regulatory goals theoretically predicts enhanced emotional awareness and amplified emotional responses to affective stimuli. While recent studies have demonstrated the importance of internal state awareness in emotional processing (Füstös et al. 2013; Farb et al. 2012), the impact of nonregulatory self‐focused attention on emotional responses remains empirically understudied. Therefore, we examined whether emotional evaluation without a regulatory goal has an aftereffect on EEG similarly to when a goal is provided, meaning that there is a lasting effect of evaluation even after stopping evaluation.

Frontal alpha asymmetry (FAA) was used as a primary index of EEG in this study. There are a variety of EEG indexes, and many reports have been published on the relationship between EEG and emotion (Coan and Allen 2004, Hajcak et al. 2010, Liberati et al. 2015). Of these, FAA is an important index often used in basic and applied research as an indicator representing an aspect of emotion (Davidson 2004). FAA is grounded in the fact that right frontal activity is associated with avoidance behavior and negative emotions, while left frontal activity is associated with approach behavior and positive emotions. FAA, calculated by subtracting log‐transformed left frontal alpha power from log‐transformed right frontal alpha power, is an index that is positively correlated with positive emotions. This indicator has shown similar trends in the results from fMRI studies (Davidson et al. 1999, Canli et al. 1998). In addition, this indicator has been found to be reproducible in studies from numerous fields (Allen et al. 2018, Reznik and Allen 2018, Meyer et al. 2015, Smith et al. 2017). Therefore, FAA is presumed to be a valid indicator of pleasant and unpleasant emotions. It is also an index that is often used as an indicator in emotion recognition research, and if the evaluations affect FAA, it could potentially shake up the results of previous studies that use FAA as an index of emotion. Therefore, examining the effects of evaluation on FAA is important for two reasons. First, it provides evidence supporting the validity of previous emotion recognition research. Second, it demonstrates the need to consider the effects of evaluation for the estimation of more sophisticated internal states, such as the estimation of not only responses to stimuli but also attitudes toward stimuli and prior context.

The present study examined whether subjective evaluation of emotion during emotion elicitation affects FAA and whether the same attitude toward the stimulus, as during evaluation, persists even after emotion evaluation has ceased and additionally influences FAA. Two experimental conditions were set for the task: without subjective evaluation (Without Evaluation) condition and with subjective evaluation (With Evaluation) condition. The experimental group participants first conducted the With Evaluation condition, followed by the Without Evaluation condition. The control group participants conducted the Without Evaluation condition twice. Based on previous findings about self‐focused attention and emotional processing, we hypothesized that evaluation would modulate emotional processing in two temporal phases: during evaluation through enhanced attention to emotional aspects and after evaluation through sustained self‐focused attention. Specifically, we predicted that the experimental group would show greater FAA modulation than the control group in both early (300–500 ms) and later (after 500 ms) time windows, reflecting enhanced emotional processing due to increased attention to their own emotional states. In addition, based on Singh et al. 2019's results demonstrating the P300 enhancement during and after evaluation of stimulus emotionality, we aimed to examine whether these findings can be extended to the situation when evaluating one's own emotional responses. We hypothesized that, despite this methodological difference, P300 amplitude would be enhanced in both evaluation and post‐evaluation phases, indicating general enhancement of attention during emotional processing.

## 2 Methods

### 2.1 Participants

A total of 41 students with no history of psychiatric or neurological disorders participated in the experiment. One participant was excluded because an issue with the program occurred during the experiment, and normal responses could not be measured. Therefore, 40 individuals participated in the study (age 21.4 ± 2.7 years [mean ± SD], 17 female). A priori power analysis using G*Power 3.1.9.7 (Faul et al. 2007) indicated that for a two‐tailed independent samples *t*‐test with a medium effect size (*d* = 0.5), a significance level of 5%, and a power of 80%, a total of 128 participants (64 per group) would be ideal. However, due to resource and time constraints, our sample size was determined based on previous similar research (*N* = 28, 14 control; Hutcherson et al. 2005), taking into account that some data would be rejected during analysis. Participants were recruited from Waseda University, Japan. The following exclusion criteria were applied: 1) impairment of any of the senses (hearing, vision, smell, taste, and touch) or of hand tasks (i.e., vision could be corrected), 2) who declined to undergo tests to measure physiological signals, 3) any medication taken within 24 h, 4) caffeine or alcohol consumption within 12 h, 5) currently experiencing auditory or visual abnormalities that interfere with daily life, 6) significant sleep deprivation or fatigue within 12 h, 7) a traumatic event experienced in the previous month combined with feelings of distress triggered by recalling that event, and 8) current participation in another intervention study in any field. These conditions were set to control for factors that may affect EEG measurements and results of the study, including baseline emotional states and mood‐related factors, and to reduce the burden on the participants as much as possible. Finally, none of the recruited participants declined to participate on grounds of the exclusion criteria set out above.

This experiment was approved by the Waseda University Ethics Review Committee on Research Involving Human Participants and conformed to the Code of Ethics of the World Medical Association (Declaration of Helsinki). Participants were recruited through advertisements posted on physical bulletin boards within university buildings and the university's student website. The advertisements included information about participation requirements and monetary compensation. All participants signed a consent form before participating in the study, had the right to withdraw from the experiment at any time, and received monetary compensation of 1500 yen per hour for their participation. To avoid informing the participants that the purpose of the experiment was related to emotions, we explained in advance that the purpose of the experiment was to measure biological signals when presented with visual stimuli and avoided the use of the keyword “emotion” in the recruitment stage and during experimentation as much as possible. After the experiment, an explanation of the study was given.

### 2.2 Visual Stimuli and Presentation

**FIGURE 1:**
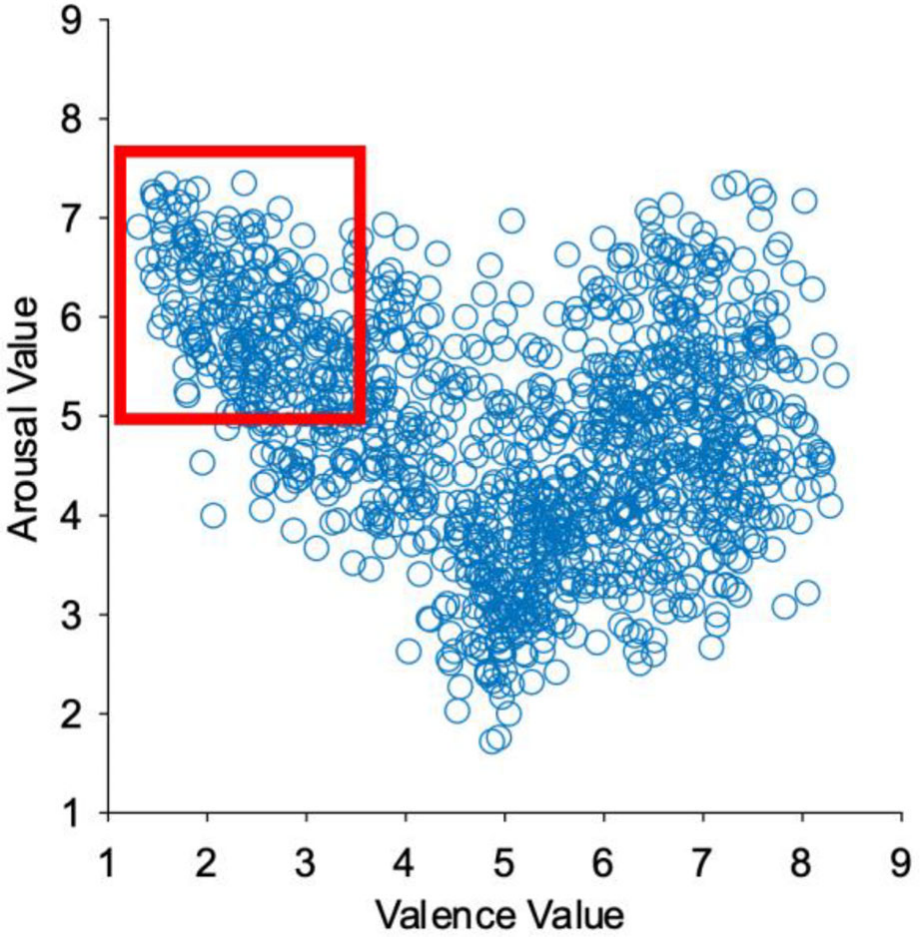
The set of pictures used for experimentation based on the scatterplot of the label values originally attached to the IAPS pictures. This figure plots the labeling values originally attached to the IAPS images, with valence on the horizontal axis and arousal on the vertical axis. Images within the red box (valence ≤ 3.55, arousal ≥ 5) were designated as negative high‐arousal images, of which 100 were randomly selected for the experiment.

The International Affective Picture System (IAPS; Lang et al. 2008) was used for the visual stimuli. These stimuli have been extensively validated using the Self‐Assessment Manikin (SAM; Bradley and Lang 1994; Morris 1995) to reliably elicit specific emotional states. A total of 134 images were extracted based on affective valence and arousal levels that were pre‐labeled by Lang et al. (2008). We extracted images that had low emotional valence (high unpleasant) and high arousal (Figure 1) based on the comprehensive meta‐analysis by Siegel et al. (2018), which demonstrated that negative high‐arousal emotions such as anger and fear produce the strongest and most reliable effects on autonomic responses. This refers to images with an arousal level of 5 or higher and an emotional valence of 3.55 or lower based on the distribution of emotional valence and arousal level. Images that were ethically problematic, such as erotic or grotesque, were visually eliminated. The threshold value of 3.55 for emotional value corresponds to the cutoff for the top 25% points, and the threshold value of 5 for arousal level is the value determined by considering the V‐shaped distribution characteristic of IAPS images for the top 25% points. Since wearing the EEG for a long period of time is burdensome for the participants, the experiment was limited to negative high arousal, which is more likely to produce a response, to shorten the experimental time. From this group of 134 images, 100 images were presented pseudo‐randomly in the same order among participants (arousal level = 5.96 ± 0.50, valence level = 2.71 ± 0.59). In the experiment, these 100 images were divided into four blocks of 25 images each. A one‐factor ANOVA analysis between blocks (Blocks 1, 2, 3, and 4) was conducted for emotional valence and arousal, and the test results showed that no significant differences were detected in the ratings of the image groups (valence: *f*[3,96] = 0.56, *p* = 0.64, arousal: *f*[3,96] = 0.28, *p* = 0.84). The mean and standard deviation of the rating values for each block are presented in Table 1.

**TABLE 1:** Rating values originally assigned to IAPS images and participant ratings for each block.

	IAPS		Participants	
	Valence	Arousal	Valence	Arousal
Block 1	2.72 ± 0.54	5.95 ± 0.68		
Block 2	2.75 ± 0.51	5.88 ± 0.50	Experimental: 3.53 ± 1.01	Experimental: 5.20 ± 1.90
Block 3	2.77 ± 0.50	6.02 ± 0.71		
Block 4	2.60 ± 0.47	6.01 ± 0.47	Control: 3.78 ± 1.19 Experimental: 3.44 ± 1.05	Control: 5.18 ± 1.85 Experimental: 5.05 ± 2.18

Visual stimuli were presented using MATLAB's Psychtoolbox (Brainard 1997; Pelli 1997; Kleiner et al. 2007) on a 13.3‐inch display with a resolution of 2560 × 1600 on a MacBook Pro. The display was positioned approximately 60 cm away from the participant. Participants wore earplugs to control auditory information and sat in a relaxed position in a chair.

### 2.3 Experimental Groups and Conditions

Participants were alternately assigned to either the experimental group or the control group based on their order of participation. Specifically, the first participant was assigned to the experimental group, the second to the control group, and this alternating pattern continued. This sequential allocation was necessary because the protocol of the control group required the response data from the experimental group participants.

**FIGURE 2:**
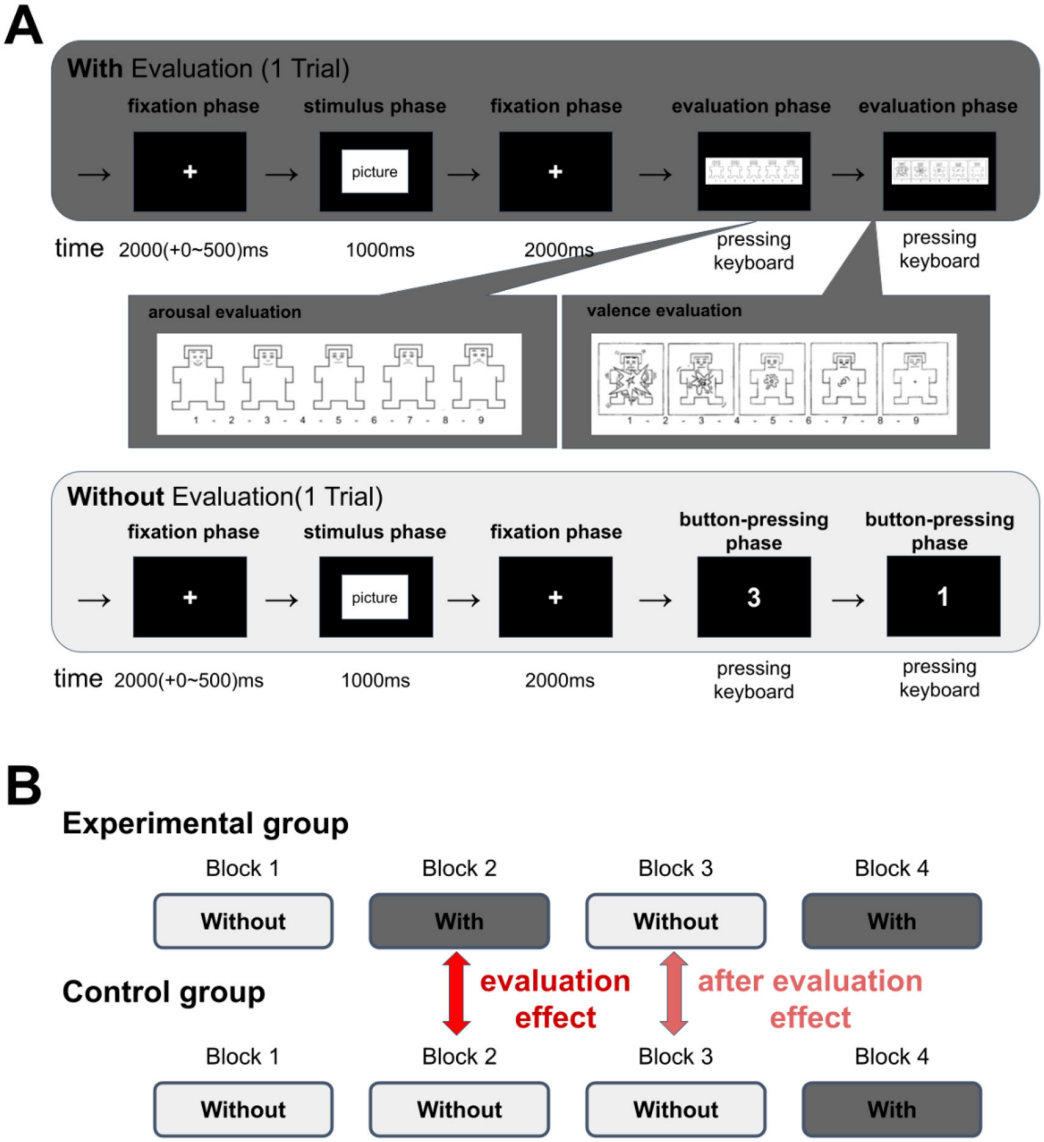
Experimental design. (A) The duration of each event for the “With Evaluation” (top) and “Without Evaluation” (bottom) conditions. The white cross was always presented except during visual stimulus presentation and evaluation. The stimulus duration was 1000 ms. The onset of the image stimulus was randomized and appeared 2000–2500 ms after the end of the previous trial. Epochs for calculating EEG indexes were extracted from the interval from stimulus to evaluation (−500 to 3000 ms after stimulus onset). (B) The order of experimental conditions in the experimental and control groups. After a practice trial for both groups under the “Without Evaluation” conditions (Block 1), the experimental group performed the trial under “With Evaluation” conditions and the control group performed the trial under “Without Evaluation” conditions (Block 2). Both groups then performed the trial under “Without Evaluation” conditions (Block 3), followed by “With Evaluation” conditions (Block 4); Block 2 was compared across the groups (control group: “Without Evaluation”, experimental group: “With Evaluation”) to examine the effects of the evaluations, and the Block 3 was compared across the groups (control group: “Without Evaluation”, experimental group: “Without Evaluation”) to examine the aftereffects of the evaluations.

The experiment was conducted under two conditions: a “With Evaluation” condition in which participants were asked to make a subjective evaluation of their own emotional state (both arousal and valence) after the presentation of each image, and a “Without Evaluation” condition in which participants were not asked to make a subjective evaluation of their emotional state (Figure 2A). The experiment was conducted for a total of 4 blocks (Figure 2B). First, a “Without Evaluation” session was conducted in both groups to fully habituate the participants to the stimuli (Block 1). Then, the experimental group completed the trial under “With Evaluation” conditions, and the control group completed the trial under “Without Evaluation” conditions (Block 2). Both groups then participated in a trial under “Without Evaluation” conditions (Block 3). Since the control group only underwent the trial under “Without Evaluation” conditions and there was no reference evaluation value to confirm whether the expected emotion was actually elicited or not, a trial under “With Evaluation” conditions was conducted in both groups at the end to collect the evaluation values (Block 4).

### 2.4 Procedure

The “With Evaluation” condition involved an initial fixation phase in which participants focused on a white cross (randomized from 2000 to 2500 ms based on MATLAB's rand function) (Figure 2A). Next, the stimulus phase consisted of a 1000 ms presentation of the IAPS emotion‐evoked image stimulus. After a 2000 ms fixation phase, in the evaluation phase participants were presented with the Self‐Assessment Manikin (SAM; Bradley and Lang 1994) and were asked to select “the one that best corresponded to their current state” using a 9‐point scale from 1 to 9 for both arousal and valence. First, the participants evaluated “arousal” by pressing the corresponding keyboard buttons from 1 to 9 and then moved to the “valence” screen. After both evaluations were completed, participants moved on to the next trial. A total of 25 trials were conducted in each condition. Our experimental design followed the paradigm used in previous studies (Mehmood and Lee 2015; Frantzidis et al. 2010). Immediately prior to the first “With Evaluation” condition (experimental group Block 2, control group Block 4), a practice trial of the “With Evaluation” condition was conducted for three trials, including instruction. At that time, when the SAM was displayed, participants were instructed to “intuitively choose the doll that most closely resembles your current state”. Participants were allowed to freely blink throughout the experiment.

The “Without Evaluation” condition was based on the literature (Singh et al. 2019) and involved the manipulation of a simple button‐pressing task instead of the emotion evaluation task. Under the “Without Evaluation” conditions, a 2 s fixation phase after IAPS stimulation was followed by a button‐pressing phase (Figure 2A). During the button‐pressing phase, participants were asked twice to press a key on the computer corresponding to a number from 1 to 9 displayed on the screen. The numbers were presented in a predetermined random order and in the same way for all participants, but in the “Without Evaluation” condition for the control group in Block 2, the numbers were used that had been answered in the “With Evaluation” condition in Block 2 for the experimental group, which took place one participant before the control group. A simple button‐pressing phase under “Without Evaluation” conditions and number control in Block 2 was set up for the purpose of controlling for keypress myoelectricity and button spatial search that were not relevant to the evaluation between groups.

The 100 selected images were presented to the participants in 25 trials × 4 blocks in the same order in a pre‐randomized permutation. This ensured that the images were always novel to the participants. Table 1 shows the mean and standard deviation of the IAPS ratings and the ratings by the participants for each group of images in each block. The participant rating was calculated by averaging the rating values for the 25 images recorded in each block, which was then used as the rating value for each participant.

Participants were provided information about the number and length of experiments but no information about the conditions or order of conditions prior to the experiment. When a condition was new, they practiced three trials prior to its implementation. That is, just before performing the experiment under “With Evaluation” conditions, participants were presented with the SAM for the first time and instructed to select the one that was most equivalent to their current state. During the practice trials, participants were presented with three IAPS images that were not included in the test blocks. These three images were common to all practice trials. Before the start of each block, resting EEG measurements were taken for 90 s each in the order of open and closed eye to establish baseline activity and control for block‐to‐block variations in participant's state. Each block lasted approximately 4 min. In between each block, there was a 1–5 min break during which the participants were not required to do anything specific, allowing them to reset their temporary emotional states. This experimental design followed established procedures (Singh et al. 2019) to control for temporal factors such as fatigue and adaptation effects. Participants were briefed about the experiment for 10–15 min before the experiment.

### 2.5 EEG Preprocessing

All data preprocessing and analysis were performed using custom‐written MATLAB scripts and EEGLAB, an open‐source toolbox for EEG data analysis (Delorme and Makeig 2004).

EEG signals were acquired using an EEG system with 29 scalp electrodes, using active dry Ag/AgCl electrodes (Quick‐30; Cognionics, San Diego, CA, USA). Electrode positions were arranged according to the international 10–20 system. Data from the left earlobe were measured for later offline reference. Signals from all electrodes were sampled at a rate of 500 Hz.

For preprocessing of the data, a finite impulse response filter of 1–30 Hz was applied to the data of each block. The continuous time series data was epoched from −1000 to 3500 ms relative to stimulus onset. The analysis target period was set from −500 to 3000 ms. This window was determined based on two considerations: the baseline period (−500 to 0 ms) followed standard practice in emotion research (Neal and Gable 2019; Singh et al. 2019), while the poststimulus period was extended to 3000 ms to capture LPP effects, which can persist up to 3000 ms following emotional stimuli (Hajcak et al. 2010). The extended epoch window (−1000 to 3500 ms) included the additional ±500 ms padding required for wavelet analysis in event‐related spectral perturbations (ERSP) calculation (Delorme and Makeig 2004). Following standard EEGLAB procedures (Delorme and Makeig 2004), at each electrode, the clean_artifacts function from the clean_rawdata plugin was used with the following parameters: flatline criterion = 5 s, channel criteria = 0.8, line noise criterion = 4, and burst criterion = 20. While most preprocessing steps followed standard EEGLAB parameters, line noise removal using pop_cleanline was performed with optimized parameters proposed by Miyakoshi et al. (2021), who identified limitations in the default settings: tau = 100, window size = 4 s, and step size = 1 s. Noisy epochs were then removed semiautomatically. To be precise regarding epoch rejection, a probability threshold of six standard deviations for a single channel and two standard deviations for all channels was applied, in addition to an amplitude threshold of −500 to 500 µV. Probability thresholding was performed many times until no data outside the threshold was detected. Independent component analysis (the runica algorithm with the extended infomax option) was performed on the epoched data. To ensure objective artifact rejection, components representing artifacts were automatically identified using the Multiple Artifact Rejection Algorithm (MARA; Winkler et al. 2011). P8 electrode data from 10 participants were not recorded due to a hardware misconfiguration, but we treated them in the same manner as noisy or poorly measured electrodes (removed in preprocessing). The data from nine participants were removed due to severe baseline noise and blink‐related noise in almost all trials. The final analysis consisted of clean epoch data from 31 participants (experimental group: 11 male/3 female participants, 21.86 ± 4.22 years; control group: 9 male/8 female participants, 21.06 ± 0.97 years). The mean number of trials per condition remaining after artifact correction was 18.71 ± 2.61 (mean ± SD) for the experimental group and 17.75 ± 3.18 for the control group. On average, 3.02 ± 1.81 (mean ± SD) electrodes were removed per participant using the clean_artifacts function.

### 2.6 FAA/ERSP Analysis

FAAs were calculated based on ERSPs, which are the results of time–frequency analysis. While we also conducted conventional FFT and STFT analyses that yielded similar results, we chose to use ERSP analysis for its ability to provide more detailed temporal resolution of alpha power changes. ERSPs were calculated for each EEG epoch of F3 and F4 electrodes based on EEGLAB's pop_newtimef with a sampling rate of 17.5 Hz, and baseline correction was made by subtracting the mean value of −500 to 0 ms of stimulus onset; the data were converted to left–right differences by log(ERSP_[F4]) − log(ERSP_[F3]). Frequency power was normalized by this calculation (Sikka et al. 2019). The waveforms for each block (Block 2 and Block 3) in the experimental and control groups were visualized by calculating the average amplitude of 8−13 Hz. We applied the most common formula used in previous studies for FAA, FAA = log(F4) − log(F3) (Vincent et al. 2021).

We examined the effects of evaluations on FAA by conducting Welch's *t*‐test on FAA in Block 2 (With/Without Evaluation) between the experimental and control groups. In addition, Welch's *t*‐test was conducted on the FAA in Block 3 (Without/Without Evaluation) between the experimental and control groups to examine the aftereffect of the evaluation on FAA. The effect size of Cohen's *d* value was calculated to show the difference between the groups.

### 2.7 ERP Analysis

ERPs were calculated at the EEG epoch of the Pz electrode and corrected using a reference to the pre‐stimulus baseline of −100 to 0 ms. The time window for identifying the P300 peak was set between 300 and 500 ms according to a previous ERP review paper (Olofsson et al. 2008) and an article calculating ERPs for similar stimuli (Cano et al. 2009). The average peak amplitude for each participant was obtained by averaging the data over the time interval.

## 3 Results

### 3.1 Induction of Negative Emotion by IAPS

**FIGURE 3:**
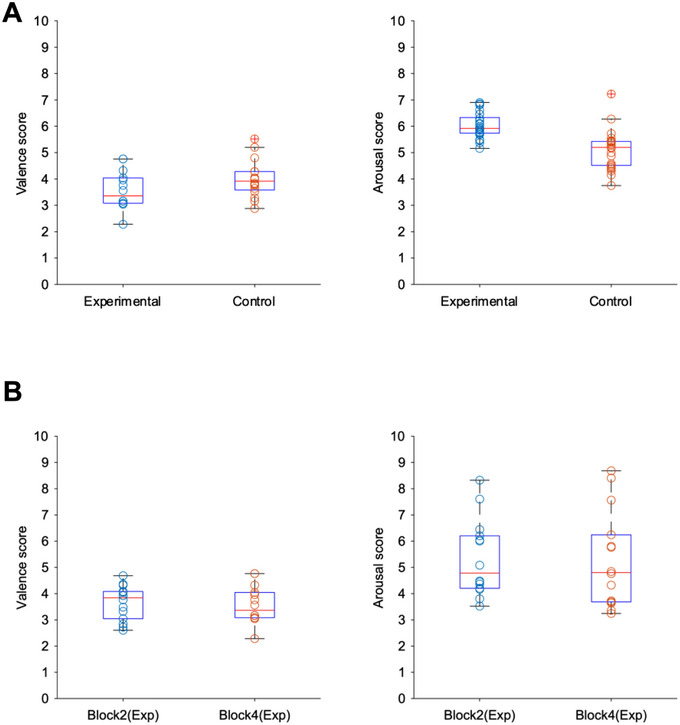
Comparison of participant evaluation values in the experimental and control groups, Block 2 and Block 4. Left: emotional values. Right: Arousal level. (A) Box plots of the average emotional valence and arousal level per participant in Block 4 for the experimental and control groups. No significant differences were detected in emotional valence or arousal (*p* > 0.05). (B) Box plots of the average emotional valence and arousal level per participant in Block 2 and Block 4 for the experimental group. No significant differences were detected in emotional valence or arousal (*p* > 0.05).

Before starting the EEG analysis, we examined participant's emotional responses to the stimuli. While we selected negative high‐arousal images from IAPS, participant's ratings indicated negative valence with moderate arousal levels, possibly due to continuous exposure to similar emotional content. Most importantly, we confirmed that similar emotional states were induced across groups and blocks (Figure 3, Table 1).

In Block 4, participants consistently rated the stimuli as negative (significantly below the neutral point of 5) with moderate arousal levels (*t*[24] = 12.29, *p* < 0.001, *d* = 3.48; arousal: *t*[24] = −0.42, *p* = 0.68, *d* = −0.12). Welch's *t*‐tests comparing the experimental and control group's ratings in Block 4 confirmed no group differences in emotional experience (valence: *t*[30] = −1.02, *p* = 0.31, *d* = −0.36; arousal: *t*[30] = −0.34, *p* = 0.73, *d* = −0.12). Similarly, within the experimental group, comparisons between Block 2 and Block 4 showed no significant differences (valence: *t*[26] = 0.26, *p* = 0.79, *d* = 0.10; arousal: *t*[26] = 0.07, *p* = 0.94, *d* = 0.03), indicating consistent emotional responses across blocks.

### 3.2 FAA/ERSP

The FAA results are shown in Figure 4. Figure 4A shows the time–frequency representation of frontal asymmetry across a broad range of frequencies (3–30 Hz), calculated as the difference between right (F4) and left (F3) power, for each participant group (experimental/control) and block (Block 2 / Block 3). Figure 4B depicts the topography of alpha power for each participant group (experimental/control) and block (Block 2/Block 3). Figure 4C plots the average time course of FAA across participants for the experimental group (blue) and control group (orange) in Block 2 and Block 3, with 95% confidence intervals. On the horizontal axis, time 0 is the stimulus onset. Our main hypothesis concerning differences in FAA is that differences are due to the presence or absence of emotional evaluation and experience. Since only negative stimuli are presented in this experiment, the more the emotion was induced by the evaluation, the lower the FAA value would be. Additional analyses using conventional FFT methods showed similar patterns (see Supporting Information). Welch's *t*‐test of the mean FAA between the groups during the 0–1000 ms stimulus interval revealed that FAA was significantly lower under the “With Evaluation” conditions of the experimental group (Block 2) (M ± SD = −0.09 ± 0.16) than under the “Without Evaluation” conditions of the control group (Block 2) (M ± SD = 0.03 ± 0.11) (*t*[23.75] = −2.55, *p* = 0.02, *d* = −0.95). No significant difference was identified for the after effect of the evaluation (Block 3) (experimental: M ± SD = 0.03 ± 0.11; control: M ± SD = 0.03 ± 0.14; *t*[29.00] = −0.19, *p* = 0.85, *d* = −0.07) (Figure 4D).

FIGURE 4Comparison of FAA between experimental and control groups. Left: evaluation effect (Block 2), right: after‐evaluation effect (Block 3). (A) Time–frequency heat maps showing the temporal evolution of FAA (log (right alpha power) − log (left alpha power)) across frequencies (3–30 Hz) for experimental and control groups in Block 2 (left) and Block 3 (right). The maps were generated using Morlet wavelet analysis and averaged across participants within each condition. (B) Line plots of FAA time variation were calculated based on ERSP analysis for the experimental and control groups in Block 2 and Block 3, with 95% confidence intervals. (C) The topography of alpha powers were derived from the alpha power of each channel within the 300–500 ms time window, obtained through ERSP analysis, for each participant group (experimental/control) and block (Block 2/3). The alpha power at each location was calculated by taking the logarithm of the alpha power and subtracting the logarithm of the average alpha power in the whole brain. The alpha power was then averaged across participants for each group. (D) Box plots of FAA for each group in Block 2 and Block 3 at the stimulus interval of 0–1000 ms. There was a significant difference in FAA between the experimental and control groups in Block 2 (*p* < 0.05).

**Figure:**
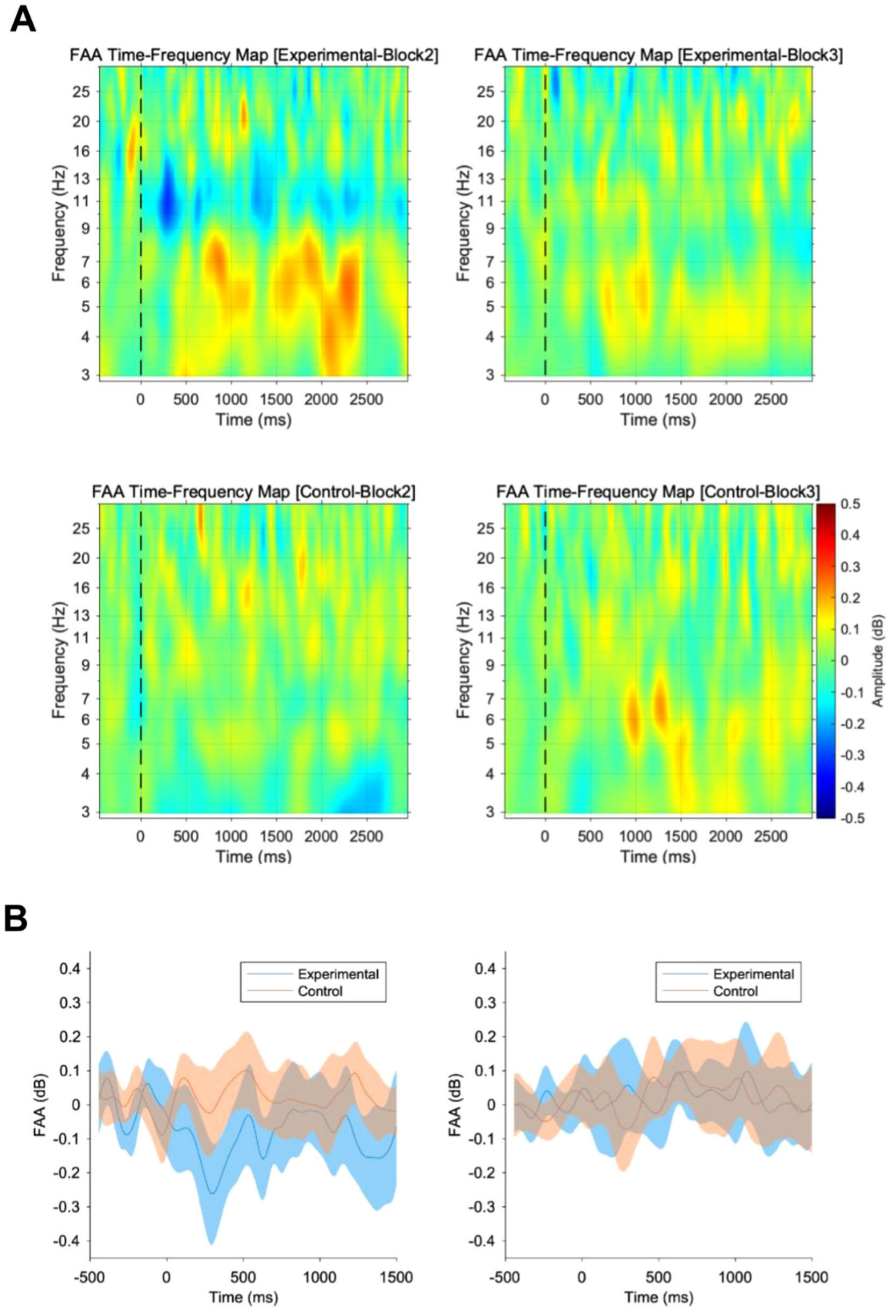

**Figure:**
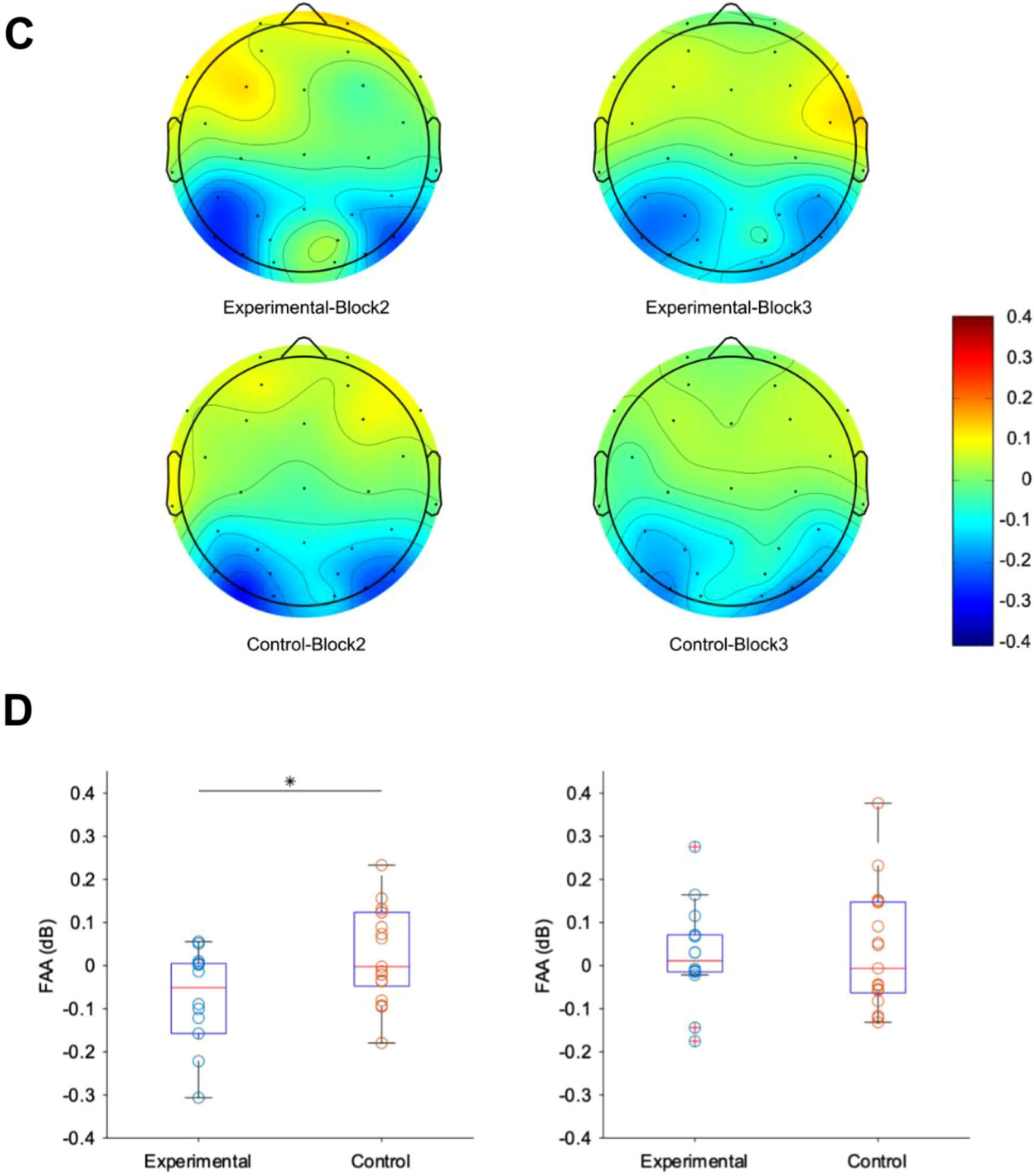

### 3.3 ERP

**FIGURE 5:**
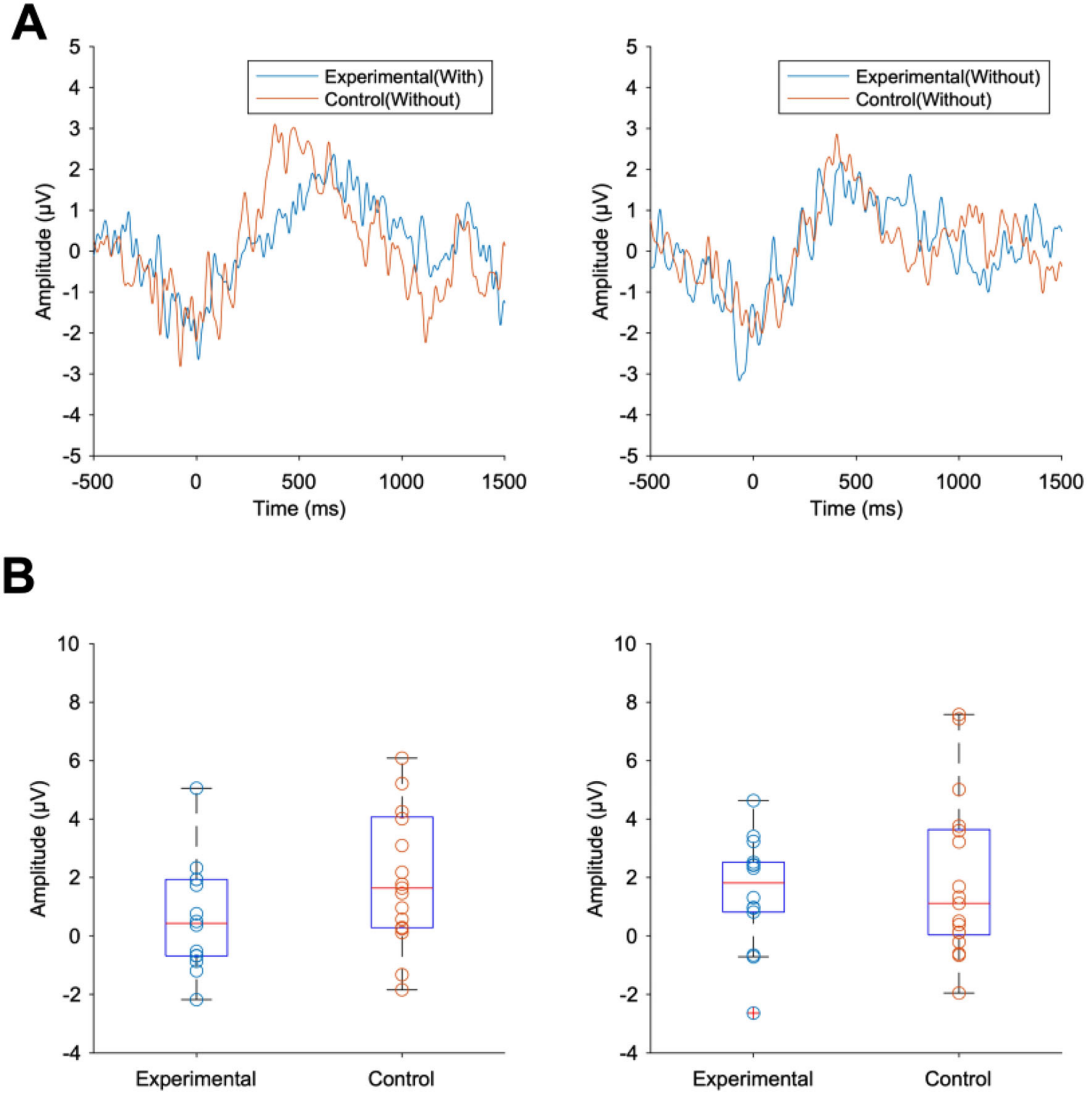
Comparison of ERP waveforms and P300 amplitude and latency between experimental and control groups. Left: evaluation effect (Block 2), right: after‐evaluation effect (Block 3). (A) The plot of time variation of amplitude in Block 2 and Block 3 for each group was calculated based on the ERP analysis. (B) Box plot of P300 shows averaged amplitude during 300∼500 ms after the stimulus in Block 2 and Block 3. There were no significant differences (*p* > 0.05).

The ERP waveforms in Pz, in which 0 is stimulus onset, are shown in Figure 5. Figure 5A shows the average waveforms across participants in the experimental and control groups for Block 2 (left) and Block 3 (right). Group comparisons of P300 amplitude revealed no significant differences between the experimental and control groups in either Block 2 (experimental: M ± SD = 0.61 ± 1.87; control: M ± SD = 1.80 ± 2.25; *t*[27.94] = −1.58, *p* = 0.12, *d* = −0.57) or Block 3 (experimental: M ± SD = 1.50 ± 1.91; control: M ± SD = 1.91 ± 2.79; *t*[28.20] = −0.48, *p* = 0.63, *d* = −0.17).

## 4 Discussion

The purpose of this study was to determine whether the subjective evaluation of one's emotional state has an effect on EEG‐measured FAA during emotion elicitation by visual stimulus and if there is an aftereffect. In the experiment, two conditions were applied: a condition in which participants were required to make an evaluation of their emotions after stimulus presentation and a condition in which no evaluation was required. The results of the experiment confirmed that FAA was significantly lower under the “With Evaluation” conditions, specifically around 300 ms after stimulus onset. No aftereffects of FAA were identified.

Our main hypothesis was that FAA underwent greater modulation under the “With Evaluation” conditions than under the “Without Evaluation” conditions due to enhanced emotional processing through increased attention to one's own emotional states. We also hypothesized that the aftereffect of evaluation existed. The results supported the hypothesis that FAA is amplified by the evaluation, but the effect does not continue after the session. In addition, an analysis was conducted in this experiment to determine if modulation was confirmed for P300, as in the study by Singh et al. (2019) during the evaluation of emotion presented in the stimulus. No amplification of P300 was observed; this dissociation between our results and Singh et al. (2019) suggests that directing attention to one's own emotional states (self‐focused attention) engages different neural mechanisms than directing attention to the emotional aspects of stimuli (stimulus‐focused attention).

The observed reduction, i.e., amplification in the negative direction, in FAA during evaluation can be interpreted from two theoretical perspectives. From an emotional valence perspective, this modulation suggests enhanced negative emotional processing during evaluation. The specificity of FAA changes to the evaluation condition, while no changes were observed in non‐evaluation conditions, might reflect heightened emotional experience when actively attending to one's feelings. This interpretation aligns with previous findings showing that self‐focused attention can amplify emotional experiences (Scheier and Carver 1977). Alternatively, from a motivational perspective, the FAA modulation might reflect the engagement of attention‐based regulatory processes. Recent studies have demonstrated that FAA not only indexes emotional valence but also reflects motivational aspects of emotional processing, with greater relative right frontal activation associated with behavioral inhibition system activation (Kelley et al. 2017; Lacey and Gable 2022). This pattern suggests that self‐focused attention during evaluation activates the behavioral inhibition system as part of a broader regulatory response, rather than simply enhancing negative emotions. Furthermore, the absence of FAA changes in non‐evaluation conditions, where participants were repeatedly exposed to negative stimuli, supports the idea that evaluation specifically triggers these motivational processes.

Figure 4A,B shows that the difference in FAA was more pronounced around 300 ms. After the onset of the stimulus, coinciding with the time window associated with attention‐related processing (Hajcak et al. 2010). This relatively early appearance of differences in FFA suggests that evaluation affects primary emotional processing, including preparation/readiness toward the stimulus, rather than slow, higher‐order processing. This timing aligns with current understanding of emotion regulation processes, where attentional deployment represents one of the earliest forms of emotional response modulation (Thiruchselvam et al. 2011). This quick and transient difference may not be identified by fMRI studies with lower temporal resolution. Regarding the aftereffect, no difference was observed, which was different from Muta and Koshikawa (2015), who reported that answering the Positive and Negative Affect Schedule (Watson et al. 1988) in advance influenced the reaction time of a subsequent emotional word discrimination task. This result may suggest that either there was a difference in reaction time but not in brain activity in the previous research, or that the aftereffect did not occur in the first place in the present research.

Our findings of enhanced emotional processing during evaluation are consistent with previous neuroimaging research. fMRI studies have shown that brain regions associated with emotion are fundamentally altered by observing and evaluating the emotion itself. Lee and Siegle  (2012) summarized fMRI studies and reported that the brain regions associated with emotion evaluation are the amygdala, lateral prefrontal cortex, and dorsomedial prefrontal cortex. The amygdala and prefrontal cortex are brain regions that have been reported to be related to emotion itself (Davidson et al. 1999, Canli et al. 1998), and thus the results of the study by Lee and Siegle (2012) suggest that emotion evaluation may alter emotion itself. Particularly relevant to our focus on self‐evaluation, Hutcherson et al. (2005) reported that the activation of the anterior cingulate and insula was increased when the participants were asked to continuously evaluate their own emotions while watching an emotion‐evoking video, compared to when the participants were asked to simply watch an emotion‐evoking video. These fMRI findings provide complementary evidence to our EEG results, suggesting that evaluation processes actively modulate emotional processing across different neural measures.

While fMRI research has investigated the effects of emotion evaluation, this theme has been overlooked by the increasing number of EEG‐based emotion classification studies over recent years. Interestingly, Brouwer et al. (2015) emphasize the need to conduct emotion evaluation during the presentation of emotional stimuli to confirm that the expected emotion is being properly recalled. However, our results indicate that such evaluations are not methodologically neutral, showing a critical difference in EEG indices between measurements in an experiment where evaluation is done and measurement in daily life where evaluation is not. This methodological concern is particularly relevant given our understanding of how evaluation processes influence emotional experiences. Muta and Koshikawa (2015) reported that answering the Positive and Negative Affect Schedule (Watson et al. 1988) questionnaire in advance influenced the reaction time of a subsequent emotional word discrimination task. They argue that evaluation increases attention to internal states and enhances self‐focused attention. These attention‐related effects of evaluation align with both our observed early modulation of FAA and current perspectives on emotion regulation processes, suggesting that our evaluation task may similarly enhance self‐focused attention and lead to a state of enhanced emotional experience (Scheier and Carver 1977).

In this study, we showed that subjective evaluations of emotions elicited a significant decrease in FAA in the negative stimulus condition. In Figure 4C, a typical decrease in occipital alpha activity during visual stimulus presentation is observed (Aranibar and Pfurtscheller 1978). Simultaneously, in Block 2 of the experimental group, an increase in alpha activity in the left prefrontal region, distinct from the occipital alpha source, is evident. This pattern of alpha activity modulation resulted in a significant decrease in FAA in the negative stimulus condition during evaluation. While this finding could reflect enhanced negative emotional processing, it may also indicate the activation of the behavioral inhibition system during self‐evaluation, as greater relative right frontal activation has been associated with behavioral inhibition (Lacey and Gable 2022). Many studies examining the relationship between brain activity and emotion are targeting evaluations of the emotion that the stimulus possesses (Gorno‐Tempini et al. 2001, Williams et al. 2006). In contrast, our current study focuses on the evaluation of one's own emotions during the presentation of emotional stimuli, from the perspective of applying estimation techniques to emotions experienced in everyday environments. Lee and Siegle (2012) reported that there are differences in the relevant brain regions when evaluating an emotion presented in the stimulus or a self‐emotion evoked by the stimulus. However, our results regarding the reduction of FAA with emotion evaluation imply a relative right frontal activation with emotion evaluation, which is consistent with those of Gorno‐Tempini et al. (2001), who reported right frontal activity during a task to evaluate facial expressions (emotions) in a facial image, suggesting common neural mechanisms might underlie different types of emotional evaluation processes.

The results for ERPs showed no significant modulation with respect to P300 amplitude. There are two possible explanations for the difference in P300 modulation between our results and those of Singh et al. (2019). One explanation is that the differences are due to whether participants evaluated the emotion presented in the stimuli or evaluated their own emotion elicited by the stimuli. Singh et al. (2019) confirmed that evaluating the emotions presented in the stimuli elicited attention to the stimuli, and thus an increase in ERP amplitude appeared. ERP amplitudes for emotional stimuli are reported to decrease when attention is directed to another object or space (Norberg and Wiens 2013). It is possible that in the present experiment's condition, in which participants were asked to evaluate their own emotional state, there was no difference in attention to the stimulus, as attention was directed to their own state, and the amplification of P300 did not occur. This interpretation aligns with our observation of enhanced self‐focused attention during evaluation, suggesting different attentional mechanisms for stimulus‐focused versus self‐focused emotional evaluation. Another explanation is that the emotional stimuli in this experiment were uniformly negative and high arousal, and this may have caused habituation or anticipation of the stimuli. In fact, the mean evaluations of arousal for all participants for each image did not differ significantly from the neutral evaluation of 5. As shown in the classical oddball task, ERPs show greater amplitude during deviant stimuli than during familiar and predictable stimuli (Squires et al. 1975). Therefore, we speculate that the ERP amplitudes for the stimuli in this experiment were small to begin with.

There are limitations to this study. First, only negative stimuli were used in this experiment owing to the limited time available for wearing the EEG instrument. Since positive and negative emotions may not be symmetrical, it is necessary to separately confirm whether similar changes are observed for positive stimuli in future studies. In addition, a simple button‐pressing task was performed as a condition without evaluation in this experiment. This is because we interpret that attention to the internal state is part of the act of evaluation. However, if we want to confirm the purer effect of emotion evaluation, we need to have a control condition with the operation of paying attention to the internal state without evaluating the self‐emotion. Moreover, while we implemented several controls to address potential effects of emotional states (baseline measurements and baseline‐corrected analysis), participant's mood prior to the experiment was not explicitly controlled between groups. This remains an important consideration for future research. In addition, although SAM provides a validated measure of emotional self‐evaluation, future studies might benefit from including additional measures of self‐relevance to further understand how participants process and evaluate their own emotional states. Lastly, effect‐size sensitivity analysis indicated that a larger sample size (128 participants) would be ideal for detecting medium‐sized effects (*d* = 0.5) with 80% power. While our sample size was determined based on previous research due to practical constraints, future studies would benefit from larger samples to achieve higher statistical power and potentially detect smaller effects.

## 5 Conclusion

In this study, we found that FAA was significantly lower when participants evaluated their own emotional responses to negative stimuli, with this effect occurring specifically during early stimulus processing (300–500 ms). This result highlights the possibility that subjective evaluation of one's own emotion may have an immediate effect on emotional and motivational processes, particularly activating the behavioral inhibition system through increased attention to one's internal states. The results suggest that evaluation may affect EEG during emotion recognition, and we therefore call for caution when considering study methodology in emotion recognition research.

## Author Contributions

**Masato Ito**: conceptualization, data curation, formal analysis, investigation, methodology, software, validation, writing–original draft, writing–review and editing. **Toru Takahashi**: conceptualization, methodology, writing–review and editing. **Yuto Kurihara**: methodology, writing–review and editing. **Rieko Osu**: conceptualization, funding acquisition, methodology, supervision, validation, writing–review and editing.

## Conflicts of Interest

The authors declare no conflicts of interest.

### Peer Review

The peer review history for this article is available at https://publons.com/publon/10.1002/brb3.70419

## Supporting information



Supporting Information

## Data Availability

The data that support the findings of this study are openly available in The Effect of Evaluating Self's Emotions on Frontal Alpha As at https://osf.io/ktzyn, reference number 10.17605/OSF.IO/KTZYN.
