# Supplementary material for: The Effect of Evaluating Self's Emotions on Frontal Alpha Asymmetry
Source: Brain Behav. 2025 Mar 23;15(3):e70419. doi: 10.1002/brb3.70419 (PMC11931087; doi:10.1002/brb3.70419)
Supplement: Supplementary file 1 — Supporting Information [file BRB3-15-e70419-s001.docx]

**Supplementary Materials**

Conventional FFT Analysis of FAA

Methods: We conducted additional analyses using conventional FFT methods to complement our ERSP analyses and provide connection to traditional FAA literature. Alpha power (8-13 Hz) was calculated using FFT for three overlapping time windows: -500ms to 500ms, 0ms to 1000ms, and 500ms to 1500ms relative to stimulus onset. FAA was computed as log(right alpha power) - log(left alpha power) following standard procedures.

Results: Table S1 shows descriptive statistics for FAA values across all conditions and time windows. Statistical analyses were conducted to compare experimental and control groups separately for Block 2 (evaluation effect) and Block 3 (after effect). For each block, comparisons were made across three time windows, with FDR correction applied within each block:

Block 2 (Evaluation Effect):

- Early window (-500 to 500ms): t[17.16] = -1.78, p_uncorrected = 0.09, p_FDR = 0.14, d = -0.69

- Stimulus window (0 to 1000ms): t[19.25] = -1.79, p_uncorrected = 0.09, p_FDR = 0.14, d = -0.68

- Late window (500 to 1500ms): t[25.33] = -0.37, p_uncorrected = 0.71, p_FDR = 0.71, d = -0.14

Block 3 (After Effect):

- Early window (-500 to 500ms): t[29.00] = 1.60, p_uncorrected = 0.12, p_FDR = 0.29, d = 0.57

- Stimulus window (0 to 1000ms): t[28.63] = 1.34, p_uncorrected = 0.19, p_FDR = 0.29, d = 0.48

- Late window (500 to 1500ms): t[27.31] = 0.57, p_uncorrected = 0.50, p_FDR = 0.50, d = 0.25

Note: FAA values were calculated using conventional FFT methods. Negative values indicate greater right frontal activity. Block 2 in the experimental group represents the evaluation condition, while all other blocks represent non-evaluation conditions. FDR correction for multiple comparisons was performed separately for Block 2 and Block 3: the three p-values from different time windows within Block 2 were corrected together, and separately, the three p-values from different time windows within Block 3 were corrected together.

# Table S1. Mean FAA values for each time window and condition

| Time Window | Group | Condition | FAA (M ± SD) |
| --- | --- | --- | --- |
| -500 to 500ms | Experimental | Block 2 | -0.11 ± 0.25 |
|  |  | Block 3 | 0.05 ± 0.24 |
|  | Control | Block 2 | 0.01 ± 0.11 |
|  |  | Block 3 | -0.11 ± 0.30 |
| 0 to 1000ms | Experimental | Block 2 | -0.11 ± 0.28 |
|  |  | Block 3 | 0.10 ± 0.35 |
|  | Control | Block 2 | 0.04 ± 0.15 |
|  |  | Block 3 | -0.08 ± 0.38 |
| 500 to 1500ms | Experimental | Block 2 | -0.04 ± 0.22 |
|  |  | Block 3 | 0.07 ± 0.34 |
|  | Control | Block 2 | -0.01 ± 0.18 |
|  |  | Block 3 | -0.01 ± 0.33 |
